# ReticularNet: Automated Pixel-Level Segmentation of Reticular Pseudodrusen on Near-Infrared Reflectance Images by Deep Learning

**DOI:** 10.1016/j.xops.2025.101038

**Published:** 2025-12-17

**Authors:** Souvick Mukherjee, Dylan Wu, Leon von der Emde, Emily Vance, Marco Ji, Mehdi Emamverdi, Tharindu De Silva, Alisa T. Thavikulwat, Jayashree Kalpathy-Cramer, Amitha Domalpally, Catherine A. Cukras, Tiarnán D.L. Keenan

**Affiliations:** 1 Division of Epidemiology and Clinical Applications, National Eye Institute, National Institutes of Health, Bethesda, Maryland; 2 Department of Ophthalmology, University of Colorado Anschutz Medical Campus, Aurora, Colorado; 3 Wisconsin Reading Center, University of Wisconsin-Madison, Madison, Wisconsin; 4 Roche Pharmaceuticals, Basel, Switzerland

**Keywords:** Age-related macular degeneration, Deep learning, Near-infrared reflectance imaging, Reticular pseudodrusen, Subretinal drusenoid deposits

## Abstract

**Objective:**

Reticular pseudodrusen (RPD) represent an important biomarker in age-related macular degeneration (AMD) but are difficult to grade and often assessed only for presence or absence, without quantitative or spatial analysis of RPD burden. The objective was to develop and validate a deep learning model for pixel-level RPD grading on near-infrared reflectance (NIR) images, which are commonly acquired in clinical practice and the most accurate en face detection modality.

**Design:**

Deep learning model development study.

**Participants:**

Five hundred eight images of 117 eyes (70 participants) with or without RPD, over a wide range of AMD severities.

**Methods:**

The ground truth grading pipeline comprised reading center multimodal grading for RPD presence and NIR annotation with RPD contours, followed by pixel-level NIR annotation of all individual RPD lesions. The data set was split 80:20 into training and test sets. A DeepLabv3-ResNet-18 segmentation deep learning model (“ReticularNet”) was trained to perform pixel-level grading of RPD on NIR images. Its performance was compared with that of 4 ophthalmologists.

**Main Outcome Measures:**

Dice similarity coefficient (DSC); intraclass correlation coefficient (ICC) for RPD lesion number, pixel area, and contour area.

**Results:**

For pixel-level grading, ReticularNet achieved a mean DSC of 0.36 (standard deviation 0.16). This was significantly higher than the mean DSC of each ophthalmologist (0.03, 0.13, 0.19, and 0.23; *P* ≤ 0.02 for each) and of all ophthalmologists together (*P* < 0.0001). ReticularNet had ICCs of 0.44 (lesion number), 0.56 (pixel area), and 0.61 (contour area), with no significant underestimation or overestimation (*P* ≥ 0.24). These values were numerically higher than the ICCs of each ophthalmologist, who had ICC ranges of –0.08 to 0.23, –0.05 to 0.40, and –0.09 to 0.58, respectively, and significant underestimation in almost all cases. For all 3 parameters, ReticularNet’s ICC was significantly higher than that of all specialists considered together (*P* ≤ 0.02).

**Conclusions:**

ReticularNet achieved automated pixel-level grading of RPD on NIR images. Its grading was superior to that of 4 ophthalmologists, across a variety of metrics. We are making the code/models available for research use. Improved access to quantitative and spatial RPD grading should lead to improved understanding of these lesions as important biomarkers of retinal disease.

**Financial Disclosure(s):**

Proprietary or commercial disclosure may be found in the Footnotes and Disclosures at the end of this article.

Reticular pseudodrusen (RPD), also known as subretinal drusenoid deposits, are increasingly recognized as an important retinal feature in age-related macular degeneration (AMD).^1^ In contrast to soft drusen, these lipid-rich deposits are located below the retina rather than the retinal pigment epithelium.^2^^,^^3^ Their presence confers increased risk of progression to late AMD, particularly geographic atrophy (GA).^1^^,^^4^ This led to their incorporation in the updated Age-Related Eye Disease Study AMD Severity Scale.^5^ Their presence is also strongly associated with severely prolonged dark adaptation, which represents an important early functional biomarker for AMD.^6^ This is often seen in association with a thin choroid and choroidal vascular perfusion deficits on OCT angiography.^7^, ^8^, ^9^ Even after GA is present, they remain an important risk factor, as they are associated with faster GA area-based expansion, as well as faster progression to multifocality.^10^, ^11^, ^12^

It may be important to consider RPD status both quantitatively and spatially. Indeed, the areas of retina affected by RPD, as well as RPD lesion numbers, are observed to differ widely between eyes, and even between time-points in the same eye.^13^, ^14^, ^15^, ^16^ However, most studies so far have considered RPD status in a binary way only, that is, present versus absent in the retina.^1^^,^^5^^,^^12^^,^^17^, ^18^, ^19^ This is mostly because reliable quantitative or spatial grading of RPD, even on multimodal imaging by expert human graders, is considered difficult and highly laborious.

Adding quantitative and spatial information on RPD status to retinal image data sets, rather than RPD presence or absence alone, is likely to improve our understanding on this feature’s role as a risk factor. First, it would help answer whether the excess risk of late AMD and of faster GA expansion increase gradually with greater RPD burden, with a dose-response association.^1^^,^^5^^,^^12^ Second, it would address the question of local versus global associations of RPD. For example, it is unclear whether RPD presence is associated with increased risk of GA globally in the eye versus only locally, that is, with RPD acting as a specific precursor lesion for GA.^1^^,^^20^^,^^21^ Similarly, for the link between RPD status and prolonged dark adaptation, some disagreement exists on the extent of local versus global associations.^21^^,^^22^ Finally, it would assist with analyses of change over time in RPD area and location, toward understanding the evolution and life cycle of RPD.^23^

In this context, artificial intelligence approaches to automated RPD detection may be helpful, toward improved accuracy, consistency, and speed. Some previous deep learning models have demonstrated robust performance for the binary detection of RPD presence or absence in an image.^24^, ^25^, ^26^ However, very few publications have described automated approaches for the quantitative or spatial detection of RPD.^27^^,^^28^ In particular, we are not aware of any automated deep learning approaches for the quantitative or spatial detection of RPD from near-infrared reflectance (NIR) images. Near-infrared reflectance images have important advantages in this respect. They are the most accurate en face modality for the detection of RPD, though the sensitivity of NIR alone in RPD detection, in relation to detection by OCT alone, has been reported at 83%–88%.^15^ As a 2-dimensional modality, NIR imaging may be ideal for RPD quantification and for simplicity of training deep learning models, in comparison to OCT. In addition, NIR imaging usually accompanies OCT imaging, so is commonly performed in retinal clinics as part of the standard of care.

Therefore, the aim of this study was to train a deep learning model, “ReticularNet,” to perform quantitative and spatial grading of RPD, that is, pixel-level annotation, on NIR images, and to compare its performance with that of ophthalmologists. The overarching goal was to develop an open-source deep learning model for research use that can quantify RPD area and lesion count, toward improved understanding of RPD’s role as an important and quantitative biomarker of retinal disease.

## Methods

### Study Participants

The study population for these analyses was drawn from participants enrolled in a longitudinal study investigating dark adaptation in individuals aged ≥50 years, including those with and without AMD.^6^ Recruitment took place at the National Eye Institute, National Institutes of Health, Bethesda, MD, between May 2011 and March 2020. The study protocol was approved by the National Institutes of Health Institutional Review Board and the research adhered to the principles of the Declaration of Helsinki. The study is registered under ClinicalTrials.gov (NCT01352975).^6^ All participants provided written informed consent. An exploratory aim of the parent study was to assess structural biomarkers predictive of AMD progression; this formed the basis for the current analyses.

The inclusion and exclusion criteria for the parent study have been described in detail previously.^6^ In brief, eligible participants had to be ≥50 years old, without advanced AMD in both eyes, and without any active ocular disease other than AMD (e.g., glaucoma or diabetic retinopathy). The participants were separated into groups based on their AMD status and RPD status, as follows. The RPD group consisted of participants with RPD in the study eye. (The criteria used for RPD grading are described later.) The control group consisted of participants without large drusen or advanced AMD in either eye (i.e., both study eye and fellow eye with no or only early AMD). Group 1 consisted of participants with large drusen in 1 eye only and no advanced AMD in either eye (i.e., study eye with intermediate AMD and fellow eye with no/early AMD). Group 2 consisted of participants with large drusen in both eyes without any advanced AMD in either eye (i.e., study eye with intermediate AMD and fellow eye with intermediate AMD). Group 3 consisted of participants with large drusen in 1 eye and advanced AMD in the other eye (i.e., study eye with intermediate AMD and fellow eye with advanced AMD). Out of these 4 groups, any participants with RPD in the study eye were removed from the original group and placed instead in a fifth and separate RPD group. (The criteria used for RPD grading are described later.)

### Imaging Protocol

The imaging protocol for the parent study has been described in detail.^29^^,^^30^ Near-infrared reflectance, fundus autofluorescence (FAF), and spectral-domain OCT images were acquired using the Spectralis HRA + OCT system (Heidelberg Engineering). The NIR and FAF images were centered on the fovea at 768 × 768 pixels (9 × 9 mm; 30° field; 11.74 μm/pixel). The volumetric spectral-domain OCT scans of the macula consisted of 121 B-scans (496 × 768 pixels), covering a 30° × 25° field of view (≈9 mm × 7 mm). Color fundus photography (CFP) was performed using the TRC-50DX mydriatic fundus camera (Topcon Medical Systems).

### Evaluation of AMD Severity and Features

Image grading for AMD severity and features was conducted at the Wisconsin Reading Center (University of Wisconsin-Madison). The grading protocol has been described in detail.^31^, ^32^, ^33^ This included the grading of AMD severity, which was performed for each eye at each visit using the 9-step Age-Related Eye Disease Study severity scale,^34^ based on the CFP images.

### Grading of RPD

**Figure 1:**
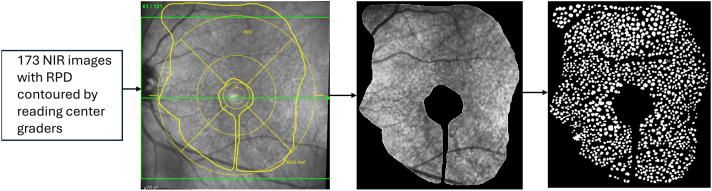
Annotation pipeline for RPD. Left to right: NIR image with RPD contour from reading center grading (39.03 mm^2^); cropped region with RPD from NIR image; binary pixel-level lesion mask. NIR = near-infrared reflectance; RPD = reticular pseudodrusen.

The pixel-level grading of RPD on the NIR images was performed in 3 steps (Fig 1). First, each eye was graded at each study visit for the presence or absence of RPD by trained graders at the Wisconsin Reading Center, on the basis of multimodal imaging, as described previously.^15^^,^^22^ Specifically, RPD were graded as definitely present in the presence of clustered subretinal deposits visible on >1 B-scan on OCT, along with corroborating features on ≥1 other modality (i.e., NIR, FAF, or CFP), with a minimum area of 0.5-disc diameters. On NIR and FAF, RPD appeared as clusters of uniformly sized dark dots, ribbon-like shapes, a net-like pattern, or a mixture of clusters and ribbons, often with bright hyperreflective spots or areas in between. On CFP, RPD appeared as pale or yellowish drusen with an indistinct, interlacing network configuration.

Second, for the instances of RPD presence, the NIR images underwent RPD contour grading by trained graders at the Wisconsin Reading Center, thereby assessing RPD area and distribution (Fig 1). Specifically, working within the Heidelberg Eye Explorer software platform (HEYEX; Heidelberg Engineering), the graders used an integrated approach based on the NIR and OCT images. They delineated the RPD contours on the NIR images but used the registered OCT images to identify the RPD borders on each B-scan, in correlation with the NIR appearance. The graders also had access to the other imaging modalities (i.e., FAF and CFP). The graders did not have access to any clinical information. The RPD contour grading was performed by a pool of multiple reading center graders, with each NIR/OCT image pair assigned to a single grader. At the end of all grading, a senior reading center grader performed verification and adjudication for all images with >20% increase or decrease in RPD area between sequential time-points of the same eye. In addition, a subset of the NIR/OCT image pairs underwent independent grading by 2 reading center graders, for an analysis of intergrader agreement. The intraclass correlation coefficient (ICC) for the RPD contour area was high, at 0.97, and the mean difference area was low, at 1.76 mm^2^.

Third, pixel-level grading of RPD lesions on the NIR images, that is, at the level of individual lesions, was performed at the National Eye Institute by a trained grader (D.W.) under the supervision of a retina specialist (T.D.L.K.), who reviewed the grading of all lesions on all images in the data set. Specifically, the RPD contours were used to isolate the relevant areas within the NIR scans and project them onto a uniform black background to standardize the region for grader annotation (Fig 1). In these contour areas, the individual RPD lesions were manually annotated at the pixel level. These annotations were converted into binary mask representations for use in subsequent image analysis. They served as the ground truth for training and evaluating ReticularNet and for evaluating the ophthalmologists.

### Data Set

The data set for these analyses comprised 508 NIR images: 173 NIR images (from 54 eyes of 34 participants) graded with RPD presence (all images used) and 335 NIR images (from 63 eyes of 36 participants) graded with RPD absence (selected randomly from all images with RPD absence). The images from the 34 RPD-positive participants were partitioned into training (∼80%) and test (∼20%) sets. This was performed at the person level to avoid data leakage, so that all images from any given participant appeared exclusively in either the training or the test set. The test set comprised 22 images, including 11 with RPD (from 11 eyes of 7 participants) and 11 without RPD (from 11 eyes of 11 participants). The training set comprised 486 NIR images, including 162 with RPD (from 43 eyes of 27 participants) and 324 without RPD (from 52 eyes of 25 participants).

### Deep Learning Model for RPD Detection and Segmentation

To develop a deep learning model to perform pixel-level grading of RPD on NIR images, we employed a DeepLabv3-based semantic segmentation model^35^ with a ResNet-18 backbone. The model (ReticularNet) was trained using NIR images paired with their corresponding manual RPD annotations as input–output pairs, with the objective of generating accurate binary segmentation masks. To optimize ReticularNet, we used a composite loss function combining binary cross-entropy and Dice similarity loss, each weighted equally (0.5). ReticularNet was trained for 10 epochs using stochastic gradient descent with momentum and a learning rate of 0.0001, implemented in Matrix Laboratory (MATLAB). Image augmentation techniques, including random reflections, rotations, translations, scaling, and shearing, were applied to increase data variability and reduce overfitting.

### Pixel-Level Grading of RPD by Ophthalmologists

To permit a comparison of deep learning performance with human specialist performance, 4 ophthalmologists performed pixel-level RPD grading on the test set of 22 NIR images, independently of each other. The 4 ophthalmologists comprised: 1 fellowship-trained retina specialist (A.T.T.), 2 ophthalmologists undertaking a fellowship in Medical Retina (M.J. and M.E.), and 1 ophthalmologist in the final year of residency who was also a certified reading center grader of retinal images (L.vdE.). Each ophthalmologist graded the full test set of 22 images. The pixel-level grading was performed using Medical Open Network for AI (MONAI) segmentation software^36^^,^^37^ deployed on a secure local server. The grading was performed on the full NIR images, so that the ophthalmologists did not have access to the reading center RPD contours. The ophthalmologists evaluated both the presence or absence of RPD in each NIR image and the spatial distribution of RPD lesions within the image, that is, pixel-level grading.

### Evaluation of Deep Learning Performance in Comparison with Ophthalmologist Performance

Performance for the detection of RPD at the image level (i.e., present or absent) was assessed by receiver operating characteristic (ROC) analysis, with area under the ROC curve as the performance metric. For each NIR image, RPD area (from either ReticularNet or the ophthalmologist grading) was used as a continuous classification score and evaluated against the binary ground truth labels of RPD presence or absence. Receiver operating characteristic analysis for ReticularNet and each ophthalmologist employed these area values: area under the curve (AUC) was computed via the trapezoidal rule as a threshold-independent discrimination metric, and a Wald test for correlated ROC curves assessed whether each area under the ROC curve differed significantly from 0.5 (random chance; *P* < 0.05).

Performance for the pixel-level grading of RPD, with reference to the ground truth grading, was assessed in 4 ways: (1) the Dice similarity coefficient (DSC), calculated as twice the number of overlapping pixels (i.e., graded with RPD in both the ground truth and the masks) divided by the total number of pixels (i.e., graded with RPD in either the ground truth or the masks); (2) agreement on the number of RPD lesions per image; (3) agreement on total RPD pixel area per image; and (4) agreement on total RPD contour area per image (defined for the ophthalmologists’ gradings as the concave hull area of all external lesion contours). Agreement was assessed by the mean ICC(2,1), a 2-way random-effects measure of absolute agreement for single observations. For each of the 4 assessments, the Wilcoxon signed-rank test was used to compare the performance of ReticularNet versus all 4 ophthalmologists considered together. In all cases, *P* < 0.05 was considered significant.

Interophthalmologist agreement was analyzed by quantifying the pairwise differences in grading between each pair of specialists. Paired Wilcoxon signed-rank tests were then used to compare each interspecialist DSC distribution, first against that specialist’s DSC with the ground truth and then against that specialist’s DSC with ReticularNet.

## Results

### Data Set

The characteristics of the study population of the parent study, stratified by AMD severity score, are shown in Table 1. Definite RPD (i.e., present on OCT and ≥1 other imaging modality) were rarely present at lower levels of the AMD severity score (in 0%–3% of instances for levels 0–5) but more commonly present at higher levels (in 8%–20% of instances for levels 6–11). In the 173 NIR images graded with definite RPD, the mean RPD contour area was 22.9 mm^2^ (standard deviation [SD] 14.2 mm^2^, range 2.2–59.9 mm^2^). In these 173 images, mean RPD contour area was generally lower at lower levels of the AMD severity score and higher at higher levels (with a maximum mean of 46.7 mm^2^ at level 7). Similarly, in these images, RPD were most commonly present in the superior quadrant only at lower levels of the AMD severity score but most commonly present in multiple quadrants at higher levels.

**Table 1:** Demographic Characteristics and Details of Reading Center Grading of RPD for all 2782 Study Visits in the Parent Study

AREDS AMD Severity Level	N Instances	Mean Age (y)	Female (%)	Definite RPD∗ N (%)	RPD on CFP† N (%)	Mean RPD Contour Area on NIR‡ (mm^2^)	NIR RPD Distribution				NIR RPD Pattern		
							S (%)	N (%)	T (%)	MQ (%)	Dots (%)	Ribbon (%)	Mixed (%)
0	394	75.3	48.0	10 (2.5)	0 (0.0)	13.2	90.0	0.0	0.0	10.0	40.0	30.0	30.0
1	149	70.1	51.0	0 (0.0)	0 (0.0)	0.0	-	-	-	-	-	-	-
2	241	71.5	49.8	0 (0.0)	0 (0.0)	0.0	-	-	-	-	-	-	-
3	101	69.1	32.7	1 (1.0)	0 (0.0)	2.6	100.0	0.0	0.0	0.0	100.0	0.0	0.0
4	287	71.7	63.4	4 (1.4)	0 (0.0)	6.8	100.0	0.0	0.0	0.0	100.0	0.0	0.0
5	156	68.1	64.1	2 (1.3)	0 (0.0)	14.9	0.0	0.0	0.0	100.0	50.0	50.0	0.0
6	309	72.5	49.8	37 (12.0)	21 (6.8)	24.7	16.2	2.7	0.0	81.1	48.6	43.2	8.1
7	402	72.6	59.5	71 (17.7)	38 (9.5)	46.7	22.5	8.5	4.2	64.8	38.0	49.3	12.7
8	171	68.2	39.8	14 (8.2)	9 (5.3)	22.8	21.4	0.0	0.0	78.6	14.3	64.3	21.4
9§	114	71.7	56.1	23 (20.2)	30 (26.3)	21.4	34.8	0.0	0.0	65.2	30.4	65.2	4.3
10‖	58	72.6	39.7	7 (12.1)	6 (10.3)	24.1	0.0	0.0	14.3	85.7	14.3	57.1	28.6
11¶	400	74.8	62.5	36 (9.0)	10 (2.5)	20.7	19.4	0.0	5.6	75.0	36.1	52.8	11.1
Total	2782	-	-	205	114	-	-	-	-	-	-	-	-

AMD = age-related macular degeneration; AREDS = Age-Related Eye Disease Study; CFP = color fundus photography; Dots = dot-shaped lesions; GA = geographic atrophy; Mixed = both morphologies; MQ = multiquadrant; N = nasal; NIR RPD Distribution: NIR = near-infrared reflectance; Ribbon = ribbon-shaped lesions; RPD = reticular pseudodrusen; S = superior; T = temporal; NIR RPD Pattern.

∗ Definite RPD N (%) = number of Instances (percentage RPD prevalence on OCTs and ≥1 other imaging modality).

† CFP RPD N (%) = number of Instances (percentage RPD prevalence on CFPs).

‡ Mean RPD Contour Area on NIR = NIR-based contour area of concave hull around the RPD lesions from reading center grading, only for images with definite RPD.

§ Noncentral GA.

‖ Central GA.

¶ Choroidal neovascularization, with or without GA.

The demographic and RPD features for the study population of 508 NIR images used in the current analyses are shown in Table 2.

**Table 2:** Demographic and Ophthalmic Characteristics of the Study Population Used for Training and Testing ReticularNet

Group	RPD Status	N Instances∗	Mean Age (y)	Female (%)	Number of RPD Lesions on NIR (Mean ± SD)	Mean RPD Contour Area on NIR† (mm^2^)	NIR RPD Distribution				NIR RPD Pattern		
							S (%)	N (%)	T (%)	MQ (%)	Dots (%)	Ribbon (%)	Mixed (%)
Total	RPD+	173	76.8	67.1	509 ± 295	22.9	25.9	4.1	2.4	67.6	31.8	55.3	12.9
	RPD-	335	75.8	62.0	0 ± 0	-	-	-	-	-	-	-	-
Train	RPD+	162	76.7	67.9	517 ± 300	23.2	25.6	4.4	2.5	67.5	30.6	55.6	13.8
	RPD-	324	76.0	62.2	0 ± 0	-	-	-	-	-	-	-	-
Test	RPD+	11	78.7	54.5	399 ± 191	18.7	30	0.0	0.0	70.0	50.0	50.0	0.0
	RPD-	11	70.3	54.5	0 ± 0	-	-	-	-	-	-	-	-

Dots = dot-shaped lesions; Mixed = both morphologies; MQ = multiquadrant; N = nasal; NIR RPD Distribution: NIR = near-infrared reflectance; Ribbon = ribbon-shaped lesions; RPD = reticular pseudodrusen; S = superior; SD = standard deviation; T = temporal.

∗ N Instances/Images = number of visits.

† Mean RPD Contour Area on NIR = NIR-based contour area of concave hull around the RPD lesions from reading center grading, only for images with definite RPD.

### Performance of ReticularNet for Detection of RPD

**Figure 2:**
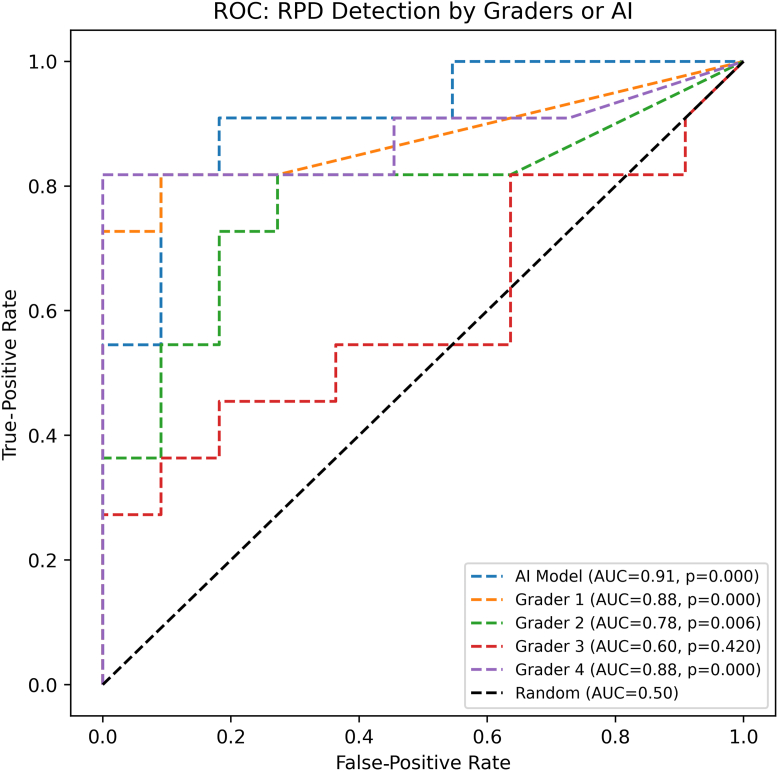
Receiver operating characteristic curves illustrating detection of RPD by ReticularNet and the 4 ophthalmologists. AI = artificial intelligence; AUC = area under the curve; ROC = receiver operating characteristic; RPD = reticular pseudodrusen.

The performance of ReticularNet and the 4 ophthalmologists in detecting RPD presence/absence at the image level was evaluated by ROC analysis (Fig 2). ReticularNet demonstrated a high level of discrimination (AUC = 0.91 [SD 0.07]), which markedly surpassed random classification (AUC = 0.50, *P* < 0.001). The levels of discrimination were high for 2 ophthalmologists (AUC = 0.88 [SD 0.08], *P* < 0.001 for each), moderate for 1 (AUC = 0.78 [SD 0.10], *P* = 0.006), and not significantly better than random classification for the other (AUC = 0.60, *P* = 0.42).

### Performance of ReticularNet for Pixel-Level Grading of RPD

**Figure 3:**
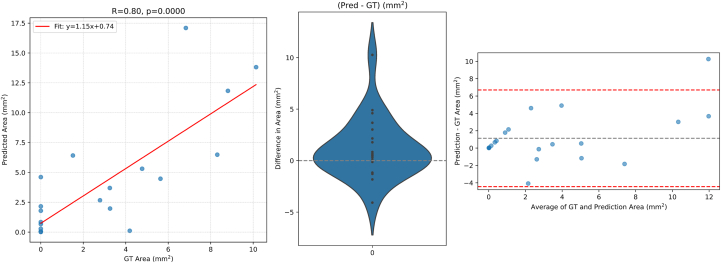
ReticularNet performance on the test set. Left: correlation between predicted and ground truth reticular pseudodrusen lesion pixel areas, with regression line y = 1.15x+0.74, R = 0.80, *P* < 0.0001. Middle: violin plot of the area error (prediction–ground truth), showing a relatively symmetrical distribution around zero. Right: Bland–Altman plot of area differences versus mean area; red dashed lines denote 95% limits of agreement (±1.96 standard deviation). GT = ground truth.

**Figure 4:**
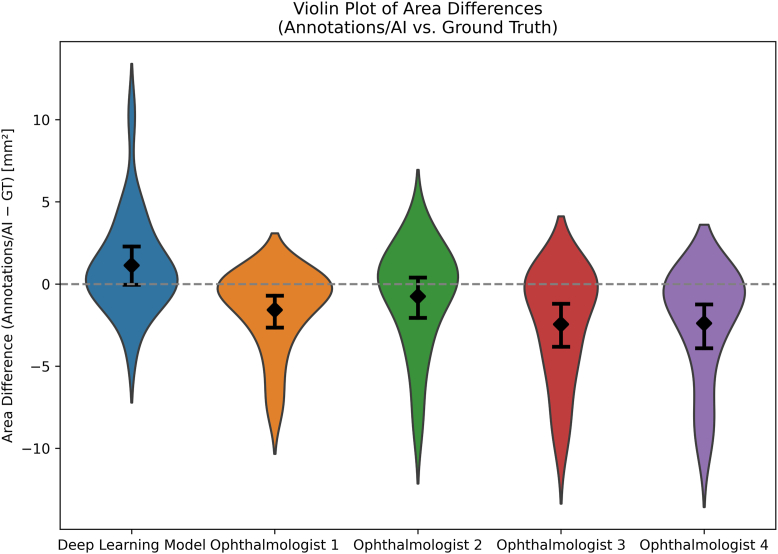
Violin plots of pixel area differences (annotation/AI prediction–GT, in mm^2^) for each method (ReticularNet and ophthalmologists 1–4). The width of each violin reflects the full distribution of per-image differences, the black diamond denotes the mean difference, and the vertical error bar shows the 95% confidence interval. The horizontal dashed line at zero indicates perfect agreement between predicted/annotated and GT areas. AI = artificial intelligence; GT = ground truth.

For the pixel-level segmentation of RPD, the performance metrics of ReticularNet and the 4 ophthalmologists on the test set are shown in Table 3 and Figure 3, Figure 4. Regarding the DSC values, ReticularNet achieved a mean DSC of 0.36 (SD 0.16). On the same test set, the 4 ophthalmologists achieved mean DSCs of 0.19 (SD 0.15), 0.23 (SD 0.14), 0.03 (SD 0.04), and 0.13 (SD 0.13). The DSC value of ReticularNet was significantly higher than that of each specialist individually (Table 3) and of all specialists considered together (*P* < 0.0001).

**Table 3:** Comparison of Deep Learning vs. Ophthalmologist Performance in Pixel-Level Grading of RPD on Near-Infrared Reflectance Images with RPD∗

Grader	Pixel-Level Comparison	**RPD Lesion Count (Mean ± SD)**	**RPD Pixel Area mm^2^ (Mean ± SD)**	**RPD Contour Area mm^2^ (Mean ± SD)**
	DSC (Mean ± SD) [*P* Value: Ophthalmologist vs. AI†]	ICC [*P* Value: Method vs. Ground Truth‡]		
ReticularNet	**0.36 ± 0.17**	**427.5** ± **214**	**6.7** ± **5.32**	**22.7** ± **14.3**
		**0.44 [0.80]**	**0.56 [0.52]**	**0.61 [0.24]**
Ophthalmologist 1	0.19 ± 0.16 [*P* = 0.005]	123.4 ± 122.1	2.2 ± 2.9	123.4 ± 122.1
		0.23 [*P* = 0.001]	0.40 [*P* = 0.001]	0.58 [*P* = 0.001]
Ophthalmologist 2	0.23 ± 0.15 [*P* = 0.02]	144.0 ± 115.1	3.0 ± 2.44	9.2 ± 7.1
		0.12 [*P* = 0.003]	0.05 [*P* = 0.07]	0.21 [*P* = 0.03]
Ophthalmologist 3	0.03 ± 0.04 [*P* = 0.005]	8.5 ± 7.8	0.3 ± 0.3	0.7 ± 0.7
		0.00 [*P* = 0.001]	0.02 [*P* = 0.001]	0.01 [*P* = 0.001]
Ophthalmologist 4	0.13 ± 0.14 [*P* = 0.002]	71.8 ± 70.6	0.6 ± 0.7	2.7 ± 2.9
		–0.08 [*P* = 0.001]	–0.05 [*P* = 0.001]	–0.09 [*P* = 0.001]
Ground Truth	-	399.4 ± 191	5.4 ± 2.79	17.9 ± 11.17
		-	-	-

AI = artificial intelligence; DSC = Dice similarity coefficient; ICC = intraclass correlation coefficient; RPD = reticular pseudodrusen; SD = standard deviation.

For each performance metric, the highest performance is shown in bold type.

∗ Dice similarity coefficient (mean ± standard deviation) is shown with *P* values comparing each ophthalmologist versus the deep learning model (second column). Mean intraclass correlation coefficient (2,1) for lesion count, pixel-based area, and contour-based area are reported as raw mean ± standard deviation, with *P* values comparing each method versus ground truth. The ground truth row lists only raw mean ± standard deviation values for each metric.

† Paired Wilcoxon test comparing each ophthalmologist’s DSC against the deep learning model’s DSC, including only RPD-positive cases.

‡ Paired Wilcoxon test comparing each ophthalmologist’s (or deep learning model’s) metric to the ground truth metric, including only RPD-positive cases.

Regarding agreement on the number of RPD lesions per image, ReticularNet achieved a mean ICC of 0.44 (*P* = 0.80), indicating moderate agreement and no significant difference in lesion numbers (with reference to the ground truth grading). By contrast, the 4 ophthalmologists demonstrated mean ICCs ranging from –0.08 to 0.23 (all *P* ≤ 0.003), reflecting poor agreement with the ground truth and significant underestimation of RPD lesion numbers. The agreement of ReticularNet was significantly higher than that of all ophthalmologists considered together (*P* = 0.001).

Regarding agreement on the total RPD pixel area, ReticularNet achieved a mean ICC of 0.56 (*P* = 0.52), indicating moderate agreement and no significant difference in pixel area (Fig 3). The 4 ophthalmologists demonstrated mean ICCs ranging from –0.05 to 0.40 (all *P* ≤ 0.07), with 3 showing significant underestimation of RPD pixel area (each *P* < 0.001), and mean biases ranging from –0.75 mm^2^ to –2.45 mm^2^ (Fig 4). The agreement of ReticularNet was significantly higher than that of all ophthalmologists considered together (*P* = 0.011).

Regarding agreement on the total RPD contour area, ReticularNet achieved a mean ICC of 0.61 (*P* = 0.24), indicating moderate agreement and no significant difference in contour area. The 4 ophthalmologists demonstrated mean ICCs ranging from –0.09 to 0.58 (*P* ≤ 0.03), with all 4 showing significant underestimation of RPD contour area. The agreement of ReticularNet was significantly higher than that of all ophthalmologists considered together (*P* = 0.018).

### Agreement between Ophthalmologists

Interophthalmologist agreement, assessed by the DSC, was generally low and variable (mean DSC range 0.02 to 0.19; Table 4). Paired Wilcoxon tests comparing interophthalmologist DSC values with ophthalmologist–deep learning DSC values indicated significantly higher agreement with ReticularNet for ophthalmologists 1, 2, and 4 (each *P* < 0.03), but not ophthalmologist 3 (*P* = 0.17), demonstrating that most ophthalmologists aligned more closely with ReticularNet than with the other ophthalmologists. A separate Wilcoxon test comparing each ophthalmologist’s aggregated intergrader DSC versus ophthalmologist–ground truth DSC yielded *P* = 0.008, showing that all ophthalmologists agreed more closely with the ground truth than with each other.

**Table 4:** Pairwise Dice Similarity Coefficients (Mean ± SD) for Reticular Pseudodrusen Segmentation among 4 Ophthalmologists, ReticularNet, and Reading Center Ground Truth∗

Annotations	Ophthalmologist 1	Ophthalmologist 2	Ophthalmologist 3	Ophthalmologist 4	ReticularNet	Ground Truth
Ophthalmologist 1	-	0.19 ± 0.14 (0.01; 0.01)	0.03 ± 0.05 (0.33; 0.95)	0.13 ± 0.13 (0.03; 0.07)	0.18 ± 0.15	0.19 ± 0.15
Ophthalmologist 2	0.19 ± 0.14 (0.01; 0.01)	-	0.02 ± 0.03 (0.6; 0.17)	0.14 ± 0.13 (0.02; 0.04)	0.21 ± 0.15	0.23 ± 0.14
Ophthalmologist 3	0.03 ± 0.05 (0.33; 0.95)	0.02 ± 0.03 (0.6; 0.17)	-	0.02 ± 0.04 (0.23; 0.74)	0.03 ± 0.03	0.03 ± 0.04
Ophthalmologist 4	0.1 ± 0.13 (0.03; 0.07)	0.14 ± 0.13 (0.02; 0.04)	0.02 ± 0.04 (0.23; 0.74)	-	0.13 ± 0.12	0.13 ± 0.13

SD = standard deviation.

∗ Parentheses show paired Wilcoxon signed rank *P* values comparing each interophthalmologist Dice similarity coefficient distribution, first against that grader’s Dice similarity coefficient with the ground truth (first value) and then against that grader’s Dice similarity coefficient with ReticularNet (second value).

### Multimodal Characterization of Segmentation Errors by ReticularNet

**Figure 5:**
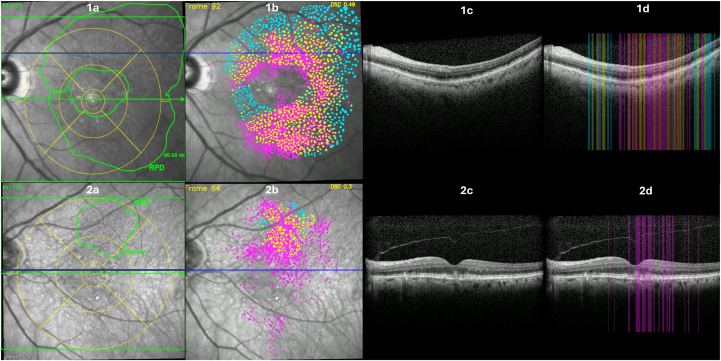
Qualitative characterization of agreement and segmentation errors by ReticularNet, by comparison with OCT, for 2 representative visits (rows 1 and 2). The top row shows fair agreement between ReticularNet predictions and ground truth, whereas the bottom row demonstrates poorer performance with multiple false-positives, likely due to drusen being misclassified as RPD in ReticularNet predictions. From left to right in each row (1 and 2): (**A**) original NIR image with overlay of RPD contour from reading center grading (green); (**B**) NIR image with overlay of ReticularNet predictions versus ground truth (yellow = agreement; cyan = false-negative; magenta = false-positive), with DSC displayed in yellow at the top right; (**C**) OCT B-scan; and (**D**) the same OCT B-scan with vertical bars color-coded for agreement (yellow), false-negative (cyan), and false-positive (magenta). DSC = Dice similarity coefficient; NIR = near-infrared reflectance; RPD = reticular pseudodrusen.

To qualitatively assess segmentation errors made by ReticularNet, we examined multiple images by overlaying the deep learning predictions and the manual annotations on the NIR images (Fig 5). We then tracked the corresponding OCT B-scans at those sites and added vertical color-coded bars to indicate concordance and discordance. In several examples, ReticularNet false-positives appeared to coincide with faint hyperreflective deposits above the retinal pigment epithelium on OCT—features that may be underrepresented on NIR—while some false-negatives occurred in low-contrast NIR regions that, on OCT, resembled non-RPD structures. Although limited in scope, these preliminary observations suggest that integrating OCT cross-validation could help contextualize and potentially refine NIR-based RPD segmentation.

## Discussion

### Main Findings and Interpretation

ReticularNet achieved automated and quantitative grading of RPD on NIR images. Importantly, NIR imaging has the advantage of being commonly performed in retina clinics and the most accurate en face modality for RPD detection.^15^ ReticularNet’s performance on pixel-level grading of RPD, assessed by the DSC, was significantly superior to that of the 4 ophthalmologists (both individually and together). We had anticipated that all DSC values would be on the lower side, given the nature of the task: tasks requiring segmentation of many features, each of small size, are prone to relatively low DSC values, because even small differences with the ground truth in the placement of the feature boundaries lead to heavy penalties with this metric. For this reason, the relative performance of ReticularNet versus the ophthalmologists is more important than the absolute performance level. In addition, the pixel-level grading of each ophthalmologist tended to align more closely with ReticularNet than the other ophthalmologists, and also aligned more closely with the ground truth than the other ophthalmologists.

Regarding the other performance metrics, ReticularNet achieved moderate agreement with the ground truth on all 3 parameters (i.e., RPD lesion number, RPD pixel area, and RPD contour area), with no significant underestimation or overestimation. For all 3 parameters, these levels of agreement were numerically higher than the agreement of each of the 4 ophthalmologists with the ground truth; they were also significantly higher than those of the ophthalmologists considered together. In almost all cases, each of the 4 ophthalmologists significantly underestimated RPD lesion number and area. Finally, although the binary detection of RPD presence/absence at the image level was not the primary purpose of this study, ReticularNet achieved a high level of discrimination (AUC = 0.91), which was numerically superior to that of each of the 4 ophthalmologists.

Of note, variability in clinician performance was observed, with ophthalmologist 3 demonstrating markedly lower metrics. This included poor discrimination of RPD presence at the image level (AUC 0.60) and very low pixel-level agreement (DSC 0.03). These results appear to represent an outlier, because intergrader agreement was consistently lower for all pairs involving ophthalmologist 3, compared with all other grader pairs (Table 4). In addition to missing some images with RPD (false-negatives), the main contributor to the reduced performance was the annotation of few RPD lesions (mean lesion count of 9). This may reflect substantial variability in ophthalmologist familiarity with the appearance of RPD.

Overall, these findings support the idea that deep learning models can be trained to generate pixel-level grading of RPD from NIR images, including lesion counts and area predictions, toward a quantitative and spatial understanding of RPD severity rather than a binary view. Importantly, both the training and test sets in this study benefited from wide diversity in the images (in terms of the wide ranges in RPD lesion number, lesion size, retinal area affected, retinal distribution, RPD type, and AMD severity level), which should lead to increased generalizability potential of the model.

### Insights into RPD and AMD Severity

In addition to permitting the development of ReticularNet, the data set provided useful information on the spectrum of RPD severity and characteristics in AMD. Although RPD extent was highly variable (range 2-60 mm^2^ contour area and 142–705 lesions), both RPD presence and extent differed strongly according to the AMD severity score. Reticular pseudodrusen presence was very rare in eyes with no to early AMD, but increasingly common at higher severity levels, corresponding to intermediate AMD to advanced disease, with a peak of 20% in eyes with GA. Similarly, mean RPD contour area was generally lower (and often confined to the superior quadrant) at lower severity levels, but tended to increase with higher AMD severity levels, with a peak of 47 mm^2^ at level 7 (i.e., high risk intermediate AMD). Lower mean areas at the highest levels, corresponding to late AMD, are likely related to RPD regression with progression to GA or neovascular AMD.^23^^,^^38^ Indeed, recent reports have observed significant decreases in mean RPD area in eyes progressing to neovascular AMD (including in the zone of neovascularization itself, but also in the zone of subretinal fluid and surrounding areas).^39^^,^^40^ As described above, the ability to generate quantitative and spatial information on RPD status is likely to improve our understanding of its role as a risk factor for late AMD and for faster GA expansion. For example, a recent genome-wide association study achieved higher statistical power by using RPD lesion number (from OCT imaging), as opposed to RPD presence/absence, as the outcome measure.^41^

### Comparison with Literature

We are aware of extremely few automated approaches for the quantitative or spatial detection of RPD from NIR images, or other en face imaging modalities, as in this study. One previous study attempted automated quantitation of the RPD contour area (within the ETDRS grid only), but, even on high-quality NIR images, model–ground truth agreement was substantially lower than ophthalmologist–ground truth agreement.^42^ Two recent studies have described automated approaches for quantitative detection of RPD from OCT images.^27^^,^^28^ One study described the development of a deep learning framework to attempt RPD quantification on OCT images from an older device (Topcon 1000), using a classification model to identify scans with drusen or RPD, followed by an image segmentation model to segment lesions as RPD or drusen.^27^ The training and testing of the segmentation model was performed at the level of individual B-scans, rather than using the full 3-dimensional nature of the OCT data. No reading center grading was performed as the ground truth, so the results were presented as a comparison of model-retina specialist agreement versus interretina specialist agreement. For the image segmentation model, the mean DSC values for RPD were 0.26 for model-retina specialist agreement and 0.42 for specialist-specialist agreement. Hence, the mean DSC of 0.26 was lower than that of 0.36 in the current study; however, direct comparison is not highly meaningful, owing to the differences in the approach and test set. Similarly, the mean ICC for RPD area was 0.61 for model–retina specialist agreement and 0.68 for specialist–specialist agreement. Overall, model performance was on the lower side, and model-specialist agreement was lower than interspecialist agreement. Some limitations of the study include: (1) the relatively small RPD data set, with only 334 B-scans with RPD (from 37 participants); (2) the exclusion of questionable cases from the data set; (3) RPD-negative B-scan sampling from eyes without any RPD at all, potentially inflating accuracy during testing; (4) RPD grading from OCT, without accompanying FAF or NIR imaging, with a single retina specialist determining RPD presence at the image and B-scan level; and (5) potential inflation of ICC values, because images with no segmentation were included as areas of zero. The lower performance may also be partially related to the older deep learning methods available at the time, as the authors used a 2-dimensional U-net, as opposed to the DeepLabv3 backbone with atrous spatial pyramid pooling used in the current study. This latter is thought to capture multiscale context better than traditional U-nets, improving accuracy especially in images with subtle color differences, as in RPD delineation.^43^

Another approach to quantitative detection of RPD from OCT images has been described recently.^28^ The authors used instance segmentation to train a deep learning model to segment RPD at the B-scan level, based on bounding boxes, segmentation masks, and a tunable threshold to generate a binary classification of an instance. Again, no reading center grading was performed as the ground truth, so the results were presented as a comparison of model–retina specialist agreement versus interretina specialist agreement. The authors reported a mean DSC value of 0.76 for model–retina specialist agreement and 0.68 for specialist-specialist agreement. However, these high values are likely partially related to the method used to calculate the DSC: during testing, at the level of each B-scan, each case of correct labeling of RPD absence (following thresholding) was awarded a DSC value of 1 for the whole B-scan. In the current study, the DSC values were derived only from NIR images with RPD present. Implementing the same approach in the current study would yield a DSC of ∼0.63 (depending on thresholding), though this approach would tend to lead to higher DSC values with higher frequency of RPD-absent instances in any test set. Other potential limitations of the study include the uniform internal data set (limited to intermediate AMD only) and the exclusion of stage 1 RPD.

### Strengths and Limitations

The main strength of this study was the existence of pixel-level annotation of RPD in the data set, for use as the ground truth, through a rigorous annotation pipeline based on multimodal imaging and reading center grading of RPD presence and contours, followed by pixel-level annotation at the level of individual lesions. This permitted both the training and testing of a deep learning model and a comparison of model performance with ophthalmologist performance. Other strengths included the wide range of RPD extents, distributions, and types, and the wide range of AMD severities, in the training and testing sets, as well as the use of multiple parameters and metrics to evaluate performance.

However, potential limitations include the size of the data set (because the performance of the model and clinicians would ideally be tested on a larger test set to ensure the results are robust and not due to chance) and the absence of external validation (because we are not aware of other data sets with pixel-level annotation of RPD on NIR images). Regarding the ground truth annotation, while intergrader reliability was assessed for RPD contour grading (ICC 0.97), formal intergrader reliability was not measured for the pixel-level annotation task itself. Although this annotation was performed within validated contours and under comprehensive specialist supervision, future studies would benefit from quantifying pixel-level annotation reliability directly. Deep learning performance may have improved further with additional training data, or with the use of a more powerful architecture (e.g., ResNet-50 or a vision transformer), and the generalizability of the model to data sets from other institutions and populations has not been verified. Finally, different software was used to produce the ground truth grading and to perform the ophthalmologists’ annotation. Although it is possible that this could have contributed to small penalties in the ophthalmologists’ performance, it appears very unlikely to be responsible for the differences in deep learning versus ophthalmologist performance. In particular, the main reason for the lower performance of the ophthalmologists was substantial undercalling of RPD areas and lesions numbers, which is unrelated to segmentation software (as opposed to accurate identification of RPD areas and lesion numbers but with small differences in the segmentation of each RPD lesion, which could have had some contribution from software differences).

## Conclusions

In summary, we developed a deep learning framework to perform pixel-level grading of RPD from NIR images, which are commonly acquired in clinical practice and represent the most accurate en face modality for RPD detection. ReticularNet was able to perform automated, quantitative, and spatial grading of RPD burden at a level exceeding that of ophthalmologists, though we recommend that its performance is tested externally, when data sets with pixel-annotation of RPD become available. We are making the code and pretrained models available, for research use only, at https://github.com/souvick91/ReticularNet. Approaches like this, which can quantify RPD area and lesion count, and provide spatial maps of RPD localization, should lead to improved understanding of these lesions as important biomarkers of retinal disease.
