# Supplementary material for: ReticularNet: Automated Pixel-Level Segmentation of Reticular Pseudodrusen on Near-Infrared Reflectance Images by Deep Learning
Source: Ophthalmol Sci. 2025 Dec 17;6(2):101038. doi: 10.1016/j.xops.2025.101038 (PMC12856432; doi:10.1016/j.xops.2025.101038)
Supplement: Supplementary Material [file mmc1.pdf]

Supplementary Material

To optimize the model, we used a composite loss function combining binary cross-entropy (BCE) and Dice similarity loss, each weighted equally (0.5).

The BCE component, derived from the categorical cross-entropy for two classes (RPD and background), is defined as:

$$L_{\mathrm{BCE}} = -\sum_{x \in X} \sum_{y \in C} w(x, y) \cdot a(x, y) \cdot logb(x, y)$$

Where:
- *X* is the set of training pixels,
- *C =* denotes the binary class labels (background and RPD),
- *a(x, y) ∈* is the ground truth label at pixel *x* for class *y*,
- *b(x, y) ∈ [0, 1]* is the predicted probability at pixel *x* for class *y,*
- *w(x, y)* is a class weighting factor to address imbalance.

Empirical class weights were applied to mitigate the imbalance between RPD and background pixels: background = 1.0, RPD = 5.0.

The Dice similarity loss, used to directly encourage spatial overlap between predicted and ground truth masks, is defined as:

$$L_{\mathrm{Dice}} = 1 - \left(2 \cdot \sum_{x} a_x \cdot b_x + \varepsilon\right) / \left(\sum_{x} a_x + \sum_{x} b_x + \varepsilon\right)$$

Where:
- a_x and b_x represent the ground truth and predicted binary masks at pixel *x*,
- ε is a small constant (e.g., 10^{-6}) to prevent division by zero.

The total loss used for training was:

$$L_{Total} = 0.5 \cdot L_{BCE} + 0.5 \cdot L_{Dice}$$

This formulation combines BCE to capture pixel-wise classification accuracy and Dice loss to enforce spatial overlap between predicted and ground truth masks. To determine the relative contribution of each component, we empirically evaluated three different weighting schemes: (0.7, 0.3), (0.3, 0.7), and (0.5, 0.5) for L_{BCE} and L_{Dice}, respectively. The (0.5, 0.5) setting yielded the most stable convergence and best validation performance across our held-out set, and was therefore used in all reported results.
